# The impact of billing/reimbursement structures on the integration of aSBI into primary care practice: implementation experiences at five different types of clinics

**DOI:** 10.1186/1940-0640-10-S2-O22

**Published:** 2015-09-24

**Authors:** Joyce Hartje, Carolyn Edney, Nancy Roget

**Affiliations:** 1 Center for the Application of Substance Abuse Technologies (CASAT), University of Nevada, Reno, Nevada, USA

## Background

The purpose of the Screen-All Project was to promote adoption of alcohol SBI (aSBI) in five primary care clinics within the University of Nevada School of Medicine Health System (System). Prior to this project, no formal aSBI activities were being conducted in these clinics or within the System. The five clinics included in the project provide healthcare services to adult men and women, including those at risk for alcohol-exposed pregnancies (e.g. women of reproductive age; college students). Two of the five clinics have non-Medicaid/Medicare/insurance funding sources for providing services, whereas three of the five clinics have Medicaid/Medicare/insurance funding sources.

The objective is to determine how billing/reimbursement impacts implementation of aSBI.

## Material and methods

Meetings were held with the medical director and manager of each clinic to gather clinic-specific information (e.g., staffing, workflow, EMR, billing/reimbursement) and identify potential barriers to aSBI implementation. This information was used to inform development of aSBI training for each clinic.

## Results

Medical personnel/staff at all five clinics participated in trainings. Four of the five clinics (two non-funding-dependent and two funding-dependent) successfully implemented aSBI. However, patient screening rates were higher at the two non-funding-dependent clinics (66% and 37%) compared to the two funding-dependent clinics (25% and 7%). Staff at the fifth clinic, which is dependent on Medicaid/Medicare/insurance funding, attended initial trainings and requested additional meetings/information but never implemented aSBI.

## Conclusions

Although all five clinics received the same training and support throughout the project, only four implemented aSBI. The two clinics most successful with the implementation were not dependent on reimbursement. One barrier to implementation for funding-dependent clinics is that Nevada's Medicaid billing codes are not turned on for conducting aSBI. Efforts have begun to get those codes activated. Lessons learned from this project could be useful when systematizing aSBI as part of routine practice in other System clinics.

